# Supplementary material for: Development of a Global Health Learning Progression (GHELP) Model
Source: Pharmacy (Basel). 2020 Dec 24;9(1):2. doi: 10.3390/pharmacy9010002 (PMC7839015; doi:10.3390/pharmacy9010002)
Supplement: Supplementary file 1 [file pharmacy-09-00002-s001.pdf]

Table S1. Codebook		
**KNOWLEDGE CODE**	**DEFINITION**	**EXAMPLE**
In general, knowledge areas regard an understanding or recognition of something.		
**Cultural AwarenessGeneral (CA-G)**	Cultural awareness refers to observing and being conscious of similarities and contrasts between cultural groups.<br><br>For the “general” subcode, their cultural awareness does not have further context to categorize what it is being applied to.	*I also feel I was able to gain a substantial amount of knowledge about the culture of Australia.*<br><br>*I was able to learn a lot about Malawian culture and their view of American culture.*
**Cultural Awareness-Health Care (CA-HC)**	Cultural awareness refers to observing and being conscious of similarities and contrasts between cultural groups.<br><br>For the “health care” subcode, students recognize how cultural similarities and differences influence care.	*Impact of economics and culture on health care*<br><br>*In addition, the cultural perceptions of HIV still affect access to care*<br><br>*I learned a lot more about the overall culture of China and how it applied in the healthcare setting*
**Health Care System – General (HCS-G)**	Students describe similarities and/or differences of the health care system without enough context to determine how it applies.	*Learning about community pharmacy and socialized medicine enhanced my understanding of the healthcare system there.*
**Health Care SystemDelivery (HCS-D)**	Students describe similarities and/or differences of the health care system within the context of the delivery of care.<br><br>Students often describe a component involved in the delivery of care (i.e. training, access to medications, provider accessibility)	*Learned how to provide care in a different health care system.*<br><br>*I saw that Ireland is about 10 years behind other European nations and the US in how their healthcare is given*
**Health Care System – Payor & Policy (HCS-PP)**	Students describe similarities and/or differences of the health care system within the context of insurance/coverage schemes or policies/regulations that influence health care.	*I learned how socialized healthcare affects pharmacy work. I learned how to process claims through a system different than the U.S.*<br><br>*During my international APPE I learned about Australia's Medicare system and the similarities and differences between their pharmacy benefit scheme and the insurancebased model in the United States.*
**Health Care System – Technology (HCS-T)**	Students describe the use (or lack of) technology.<br><br>This is typically used in the context of the health care system and often is in reference to paper charts.	*I was surprised by the use of a paper patient medical charts and lack of technology*

**Medication-Knowledge (MK)**	Students describe knowledge regarding medications (in general, specific classes, those to treat certain conditions, those specific to that country, etc.) without enough context to justify knowing about the use of the medications	*I was also able to learn new drugs that are not FDA approved medications or available in the United States.*

**Medication-Use (MU)**	Students specify learning about the use of medications beyond just general increased knowledge. Usually in the context of how they are used to treat/manage disease states.	*I feel that my knowledge of medication use has greatly expanded*
**Complimentary & Alternative Medicines (CAM)**	Students describe knowledge of complementary and alternative medications (CAM) including traditional Chinese medicine (TCM).	*One of the biggest aspects of pharmacy that I learned about was how important Traditional Chinese Medicine (TCM) is to patients in China.*<br><br>*the perception of western medicine is varies WIDELY throughout the country, as does perception of natural medicine*
**Role of the Pharmacist (ROP)**	Students describe increased knowledge of the role of the pharmacist, usually in the context of their visiting country or a specific health discipline	*I am also more aware of the limited role of pharmacists in China, but am excited that the pharmacy schools there are working to improve this.*
**Pharmacy Practice (PP)**	Students describe some aspect of pharmacy practice, usually in reference to a similarity or difference.	*from my experience, pharmacy practice in Lilongwe is still strongly based on inventory management*
**Pharmacy Education (PE)**	Students describe some aspect of pharmacy education or training.	*I also learned more about the Master's pharmacy program and hospital system in Ethiopia.*
**Disease State Knowledge (DSK)**	Students describe their knowledge of certain disease states. This can include knowledge of medications, pathophysiology, and guidelines used in the context of certain disease states.<br><br>Students often reference disease states unique to their rotation site and/or those more prevalent at their rotation site.	*I gained a lot of knowledge on disease states that I had not previously encountered on my other rotations in the US.*<br><br>*Gained knowledge of parasitic infections*<br><br>*I also learned about different acute medical conditions such as "thunderstorm asthma,"*
**Disease State Management (DSM)**	Students reference the treatments used or protocols involved in addressing disease states. This is often in conjunction with disease state knowledge, but not always.	*I gained knowledge of treating pediatric patients, specifically pediatric patients with cystic fibrosis.*
**Diagnostics (D)**	Students reference knowledge of diagnostics used to evaluate disease states and conditions.	*I learned quite a bit about interpreting an x-ray, what kinds of labs are drawn to rule out certain disease.*

**HIV**	Students mention some aspect of HIV. Usually in conjunction with TB and malaria.	*My experiences in Kenya exposed me to disease states we do not see commonly here in the US (HIV/AIDS, malaria, cryptococcal meningitis, TB, PCP, etc.).*
**Tuberculosis (TB)**	Students mention some aspect of TB. Usually in conjunction with HIV and malaria.	*My experiences in Kenya exposed me to disease states we do not see commonly here in the US (HIV/AIDS, malaria, cryptococcal meningitis, TB, PCP, etc.).*
**Malaria (MAL)**	Students mention some aspect of Malaria. Usually in conjunction with HIV and TB.	*My experiences in Kenya exposed me to disease states we do not see commonly here in the US (HIV/AIDS, malaria, cryptococcal meningitis, TB, PCP, etc.).*
**Infectious Disease (ID)**	Students mention infectious diseases such as those mentioned above.	*My experiences in Kenya exposed me to disease states we do not see commonly here in the US (HIV/AIDS, malaria, cryptococcal meningitis, TB, PCP, etc.).*
**Non-Communicable Disease (NCD)**	Students mention a non-communicable disease area (i.e. oncology, diabetes)	*I learned more about cancer pain management, lymphoma, CML, and ophthalmology topics.*
**Barriers to Care – General (BC-G)**	Students describe challenges or barriers patients face in accessing health care. These barriers can be explicitly referenced or implicitly inferred within the context of the comment.<br><br>General subcode does not allow for further interpretation	*I learned how to handle situations where we were out of medications or didn't have enough supplies to treat a patient like we normally would.*<br><br>*In addition, the cultural perceptions of HIV still affect access to care*
**Barriers to care - Resource Limitations (BC-RL)**	Students describe increase knowledge and ramifications of limited resources and how this can impact an approach to patient care	*I learned that Zambia's Teaching Hospital doesn't have enough beds and patients are placed on the floor.*<br><br>*I learned how to handle situations where we were out of medications or didn't have enough supplies to treat a patient like we normally would.*

**Barriers to Care – Supply Chain (BC-SC)**	Students describe challenges regarding medication availability or access.<br><br>Students sometimes mention their knowledge of donated medications. Comments are often in the context of limited resources and/or barriers to care.	*Challenges associated with this were that medication availability was always changing and we sometimes had to alter our treatment based on this.*<br><br>*Saw the delivery of a "Project Cure" donation and learned about how helpful or not very helpful donations like this can be.*
**Social Determinants of Health (SDH)**	Students describe a social determinant of health (socioeconomic status, access to funds, education, transportation, social norms, literacy, culture, etc.)	*I learned about the disparity between those with money and those without and how it has a direct effect on the person's medical care.*<br><br>*Prior to my international rotation, I had vague understanding of impact of social and economic issues on delivery of healthcare services, but*
		did not realize how it could manifest on the health of each individual.
**Disease Burden (DB)**	Students mention the burden of a disease.<br><br>Often in reference to epidemiology.	*I realized how large of an epidemic HIV is and broadened my knowledge in this treatment area.*<br><br>*The prevalence of HIV in Zambia is 12 percent.*

**SKILL CODE**	**SKILL DEFINITION**	**SKILL EXAMPLE**
In general, skill areas regard <u>the ability to do something</u>.		
**Communication-Patient (C-P)**	Describes the ability to communicate with patients in some capacity. Includes comments in reference to health literacy. Includes patient counseling, if applicable.	*When speaking with patients, I learned how to effectively describe medications and processes in a way that others could understand me.*
**Communication-Healthcare Personnel (C-HP)**	Describes the ability to communicate with providers (i.e. pharmacists, physicians, nurses, preceptors, etc.) in some capacity	*I definitely practiced my verbal and non-verbal communication skills by doing presentations and sharing knowledge with local healthcare professionals.*
**Communication-Interpersonal (C-I)**	Describes the ability to communicate with peers (i.e.<br>other students) in some capacity	*I feel that my general interpersonal skills were GREATLY enhanced as was my comfort in communicating with/getting to know/etc. others*
**CommunicationGeneral (C-G)**	Describes the ability to communicate without context to a particular group	*My decision-making and communication skills were greatly enhanced by this experience.*

**Communication – Cross Cultural (C-CC)**	Describes the ability to communicate within a cultural context	*My communication skills were also strengthened by learning to effectively communicate needs and key points to people in a culture slightly different than my own.*
**CommunicationLanguage (C-L)**	Describes the ability to communicate in a language besides English.	*I would say the only specific skills I gained are that my Japanese improved.*
**Patient Care - Patient Counseling (PC-PC)**	Describes the ability to educate/counsel patients.	*Many patients did not speak English, so I had to find individuals to translate for me in order to provide education. This education was an effort to help them manage their health conditions and prevent costly readmissions.*
**Patient Care – General (PC-G)**	Describes the ability to provide patient care in some capacity without further details/context.	*Treating the patients in Kenya required me to problem solve then things did not work as seamlessly as they would have in the US, and the experience forced me to take ownership of my patients and their care.*

**Patient Care- Disease State Management (PC-DSM)**	Describes the ability to manage disease states beyond describing disease state management in a knowledge context.	
**Patient Care – Clinical Skills (PC-CS)**	Describes the ability to apply clinical knowledge to a problem or issues. Could also generally mention "clinical skills"	*I enhanced my ability to communicate with patients that spoke a different language and make clinical decisions while keeping in mind their different cultural beliefs.*<br><br>*Gained experience applying drug knowledge outside of guidelines.*
**Patient Care – Chart Review (PC-CR)**	Describes the ability to review patient charts, usually in reference to paper charts.	*One new skill that I picked up was the ability to read and interpret written charts.*
**Patient Care – Compounding (PC-C)**	Describes the ability to compound, usually in a radio pharmacy setting	*I learned how to compound IV chemotherapy.*
**Interprofessional Collaboration (IC)**	Describes the ability to work and be included as part of an interprofessional team	*My ability to problem solve and work on an interdisciplinary team were enhanced during this rotation.*
**Empathy (E)**	Describes the ability the understand and share one's feelings. This is usually within the context of patient care.	*Continuing this practice in the future will make me a more empathetic and understanding provider.*

**Literature Review (LR)**	Describes the ability to search literature. Includes drug information searches.	*In the end, I gained new knowledge about particular pediatric medication issues while enhancing my skills to search literature and self-direct.*
**Problem Solving (PS)**	Describes the ability to solve problems	*My problem-solving skills were definitely improved through this experience.*
**Critical Thinking (CT)**	Describes the ability to creatively and critically think about issues. Can correlate at times with having to adjust to limited resources.	*I was forced to think outside the box on a regular basis in order to provide the best care for my patients.*<br><br>*Sometimes, this required thinking creatively and with the team to provide that care as many resources were not available.*
**Adaptability (A)**	Describes the ability to adapt to new or different situations. Can often be interpreted as flexibility.	*My adaptability skills were enhanced by my APPE due to the severely restricted resource setting we worked in.*
**Navigation (NAV)**	Describes the ability to find one's way/get around. Usually in the context of activities outside of their rotation and involving travel.	*Outside of the APPE, I learned a lot about how to make my way around a place that I have very little knowledge about.*
**Initiative (I)**	Describes the ability to take action without being told to do so. (i.e. being proactive)	*Furthermore, I also became more proactive and took initiative when I saw an opportunity for me to step in and improve care.*
**Self-Directed (SDL) Learning**	Describes the ability to direct their own learning. These are usually within the context of not having structured activities to do at their rotation site.	*There were times when I had nothing to do when I wasn't rounding or working on my designated project, so*
		I took it upon myself to look up literature on topics and issues within pediatric pharmacy.
**Patience (P)**	Describes the ability to accept or tolerate delay without getting frustrated or upset	*I think that patience, as stated before, was a skill that I greatly developed during my time in Malawi*
**Leadership (L)**	Describes the ability to lead in some capacity.	*I think I was able to rise as a leader and sharpened my "managerial" skills.*
**Reflection (REF)**	Describes the ability to self-reflect in some capacity.	*It made me more reflective of how healthcare is something that we can take for granted.*
**Future Applicability (FA)**	Describes the application of a skill in the future.	*I learned to choose my words more carefully and be more detailed in my explanations, which will serve me well both personally and professionally in the future.*

**Research (RESEARCH)**	Describes the ability to perform some aspect of research.	*I conducted an experiment with another student. I had never designed and completed a project mostly on my own before, so this definitely improved my research skills.*

ATTITUDE CODE	ATTITUDE DEFINITION	ATTITUDE EXAMPLE
In general, attitude areas regard a way of thinking or feeling about something.		
**Appreciation – US Health Care (A-UHC)**	Describes an appreciation for the US health care system.	*Overall, I feel like I have a greater respect for my healthcare system in the US*
**Appreciation- USA (A-USA)**	Describes appreciation or gratitude for US without a specific health care context.	*I am very grateful to be a US citizen and live in a free country.*
**Appreciation – US Pharmacy (A-UP)**	Describes an appreciation for US pharmacy practice and norms.	*One attitude and perspective that I definitely gained was how thankful I am that pharmacy is so progressive in the United States.*
**Appreciation – Limited Resources (A-LR)**	Describes an appreciation for the utilization of limited resources. Can describe trying to make the best out of what you have.	*I knew I was going into a country with much fewer resources than my own. However, these healthcare providers never let that get them down.*
**Appreciation – Cultural (A-C)**	Describes and appreciation for the culture one is in.	*I would say that Malawians taught me to appreciate what I have and to always find some joy in the little things I do.*<br><br>*In general, my perspective of the country as a whole was changed after spending time there.*
**Appreciation – Teamwork (A-T)**	Describes an appreciation for teamwork or being part of a team. Usually in the context of interprofessional interactions	*At the end of the day, the patients care depends on each member and his/her role, and there are important distinctions in each members*
		function, which is why teamwork is critical.
**Appreciation – Experience (A-E)**	Describes their experience as amazing, great, life changing, etc. Students can also comment on how their experience seemed to be invaluable.	*The experience was unlike any other rotation that I have had.*<br><br>*I really do feel that this was a once in a lifetime opportunity and I'm so appreciative to have had to chance to participate.*
**Point of View - General**	Describes having a new perspective or outlook on something without specific context.	
**Point of View – Open Mind (POV-OM)**	Describes having an open mind or outlook.	*That having an open mind and being flexible are keys to success.*

**Point of View–Patient Care (POVPC)**	Describes a way of thinking or feeling towards patient care due to the experience.	*I learned how important it is to keep the patient at the center of everything.*
**Point of View - Alternative Approach (POV-AA)**	Describes the thought that there is no universal "right way" to do something and that while others may do something different, it still may be best/appropriate	*To know that we are different, but one way of doing something is not necessarily better than another.*
**Eye Opening (EO)**	Uses the phrase eye opening to describe some component of their experience. Includes students commenting on their expectations being challenged. Often indicated by being "surprised" to see or experience something.	*I feel this experience was eye-opening and my perspective and attitudes are irrevocably changed for the better.*<br><br>*I did not expect some of the similarities which made me realize that although I was in a completely different third-world setting, the general process in providing healthcare is still the same*
**Respect (RESPECT)**	Describes having respect for something.	*I gained a lot of respect for countries that provide national healthcare*
**Confidence (CON)**	Describes having or being more confident<br><br>Also includes students describing how they are better off or will be a better practitioner for having this experience.	*I gained a lot of confidence through this as well.*<br><br>*I will be a better pharmacist because of what I learned during my time in Kenya.*
In general, the codes used in the OTHER section are below, but most closely align with those of attitudes		
**Recommended (REC)**	Describes how they would recommend this experience to others and/or wish that more people had the opportunity to participate	*I would choose to do an international rotation again if I were to do it all over!*<br><br>*I wish more people had the opportunity to do international rotations.*
**Inspiration (INS)**	Describes how the experience inspired them or was itself inspiring	*Observing how hard pharmacists in Australia are working to have their voice heard as a member of the healthcare team and to pioneer residency and fellowship programs was really inspiring and instilled a*
		new passion in me to work hard for myself and the profession as I move forward in my career.

**Global Health Perspective (GHP)**	Describes a perspective or outlook relating to global health	*Global health, not just US health, is important because disease does not have borders.*<br><br>*It has sparked a desire in me for global health, and I am looking for residencies that offer global health rotations.*
**Travel (T)**	Describes how their opportunity to travel in correlation with their rotation contributed to their rotational experience	*I was able to travel through Europe and visit new places and had the opportunity to live in a foreign country for 8 weeks.*
**Self-Awareness (SA)**	Describes becoming more aware about themselves	*I learned so much about myself and definitely grew as a person.*
